# Acidification by nitrogen metabolism triggers extracellular biopolymer production in an oleaginous yeast

**DOI:** 10.1128/aem.00947-25

**Published:** 2025-10-06

**Authors:** Henrique Sepúlveda Del Rio Hamacek, Oksana Tingajeva, Katharina Ostertag, Alīna Reķēna, Aleksandr Illarionov, Piia Jõul, Paola Monteiro de Oliveira, Giselle de La Caridad Martín-Hernández, Bettina Müller, Nemailla Bonturi, Volkmar Passoth, Petri-Jaan Lahtvee, Rahul Kumar

**Affiliations:** 1 Department of Chemistry and Biotechnology, Tallinn University of Technology 54561, Tallinn, Estonia; 2 Department of Biotechnology, Institute of Bioprocess Science and Engineering, BOKU University of Natural Resources and Life Sciences 27270, Vienna, Austria; 3 Institute of Technology, University of Tartu 124633, Tartu, Estonia; 4 Department of Molecular Sciences, Swedish University of Agricultural Sciences 8095, Uppsala, Sweden; Chalmers tekniska hogskola AB, Gothenburg, Sweden

**Keywords:** biopolymer, extracellular microbial biopolymer, extracellular polysaccharides, nitrogen metabolism, overflow metabolism, oleaginous yeast, *Rhodotorula toruloides*

## Abstract

The oleaginous yeast *Rhodotorula toruloides* is a natural producer of lipids and carotenoids. However, its potential as a producer of extracellular biopolymers remains unexplored. Hence, we aimed to evaluate the *R. toruloides* CCT0783 for extracellular biopolymer production. We found that the carbon-to-nitrogen ratio influenced exopolysaccharide (EPS) production, reaching 5.84 ± 0.45 g/L under glucose-rich conditions. We characterized the crude biopolymer using FTIR and GC-MS, identifying polysaccharide peaks, wherein EPS consisted of (%) glucose (88.83 ± 4.87), galactose (5.50 ± 1.50), mannose (4.80 ± 1.57), and xylose and arabinose (0.87 ± 0.04) monomers. The dried EPS also contained a fractional presence of protein (1.0%). Interestingly, inorganic, but not organic, nitrogen metabolism was associated with the acidification of the culture environment and simultaneous EPS production. A comparison of cultivation conditions revealed that EPS was produced when metabolic activity contributed to the culture media acidification to a pH of approximately 2. Finally, a comparative bioinformatics analysis allowed us to map the putative EPS biosynthesis and transport pathways, as well as regulators of intracellular pH maintenance. In conclusion, our study demonstrates *R. toruloides’* potential as an extracellular microbial biopolymer producer, enabling its consideration for extracellular bioproduction, besides its typically reported intracellular products.

**IMPORTANCE:**

Microbial biopolymers have been extensively studied for their impact on the environment and on health. However, developing a biotechnology process for producing such biopolymers remains challenging despite their potential for valuable applications. Considering this opportunity, we investigated the oleaginous yeast *Rhodotorula toruloides* as a producer of extracellular biopolymers, which, to date, is mostly used as a cell factory for intracellular bioproducts, namely, lipids and carotenoids. Our study identifies the conditions and maps pathways that allow exopolysaccharide (EPS) production in this yeast. These biopolymers, besides highlighting *R. toruloides* potential for extracellular production, could be deployed in diverse applications, from gelling agents in pharmaceuticals to emulsifiers in the food industry. Furthermore, our comparative bioinformatics analysis provides a foundational resource that could enable the development of *Rhodotorula* cell factories for extracellular bioproduction.

## INTRODUCTION

*Rhodotorula toruloides* is a basidiomycete oleaginous yeast known for its natural ability to produce carotenoids and neutral lipids (1). Additionally, it can be engineered to synthesize value-added chemicals such as fatty-acid esters, fatty alcohols, and terpenes (1, 2). In *R. toruloides*, significant advancements have been made in improving intracellular lipid and carotenoid production by optimizing cultivation conditions (3, 4), deploying metabolic engineering tools (5), or performing laboratory adaptive evolution (6). For instance, nutrient limitation conditions (nitrogen, phosphorus, and sulfur) increase lipid and carotenoid biosynthesis (4, 7). Salt stress reportedly enhances carotenoid production, wherein slight changes in culture density and a negative impact on glucose metabolism were observed compared to conditions without high salt concentrations (8).

Due to *R. toruloides’* ability to consume and grow on diverse substrates, such as crude glycerol, lignocellulosic hydrolysates, and wastewater, and to tolerate inhibitory compounds in the environment, it is considered a robust model organism for the development of sustainable processes (9–13).

In biotechnology, many microbial cell factories are based on yeast fermentation of sugars, producing extracellular biochemicals partially due to the Crabtree effect, where excess carbon overflows in metabolism are directed to fermentation products, allowing energy and redox cofactors maintenance during cellular growth (14, 15). *R. toruloides* is a Crabtree-negative yeast; therefore, aside from carbon dioxide, it does not typically produce a major byproduct metabolite in conditions with high concentrations of sugars (16, 17). However, wild-type *R. toruloides* (strains NRRL Y-1588, NRRL Y-1091, NRRL Y-6672, and NRRL Y-17902) were reported to produce slime on leaf surfaces, where a typical biofilm formation may occur in the native environment (18–20). Interestingly, in our previous study (8) on *R. toruloides* CCT0783, we observed cellular aggregate formation in laboratory conditions, which led us to hypothesize that these aggregates may form due to cell-cell interactions, potentially caused by the production of extracellular polymeric substances in this yeast. However, such secreted products have not been previously quantified in *R. toruloides* CCT0783, motivating our present study.

Yeasts, bacteria, algae, and other microorganisms can produce exopolysaccharides (EPS) as secondary metabolites (20). The EPS provides those microbes with a physiologically viable microenvironment, protecting them against exogenous stressors such as high temperature variations, low or high pH levels, UV radiation, desiccation, and oxidative compounds (21). EPS is also known to enable yeasts and bacteria to adhere to natural and synthetic surfaces through biofilm formation (22). These biopolymers, including xanthan, dextran, scleroglucan, polyhydroxyalkanoates (PHA), and poly(ester amide)s (PEAs), are deployed as emulsifiers, stabilizers, binders, gelling agents, coagulants, lubricants, thickening agents, or in the production of sustainable and biodegradable materials (21–25).

Studies have reported that the composition and yield of microbial EPS change according to environmental conditions and nutrient availability (26–28). For example, the carbon-to-nitrogen (C:N) ratio can influence EPS production in yeast, bacteria, and cyanobacteria. Carbon is used for the production of EPS building blocks and as an energy source for cellular metabolism, while nitrogen allows biosynthesis of cellular components, such as amino acids and nucleotides (29, 30), and is a crucial factor in intracellular pH and carbon flux regulation (31, 32). Interestingly, for many yeast genera, a strong acidification of the culture media is reported to occur in EPS-producing conditions, and low pH is required to achieve relevant yields, indicating the relevance of the pH value (33–35). Carbon-to-phosphorus (C:P) and carbon-to-sulfur (C:S) ratios, type of carbon source, temperature, and oxygenation are other parameters that can affect the production of microbial extracellular biopolymers (36).

Therefore, given our previous observation on cellular aggregate formation (8) and the relevance of nutrient sensing and signaling in EPS production, our study aimed to investigate the role of carbon and nitrogen metabolism in *R. toruloides* CCT0783 for potential production of extracellular biopolymers. For readouts, we deployed qualitative and quantitative approaches, characterizing yeast physiology and produced EPS using FTIR and GC-MS techniques. During the study, we identified that the pH of the media decreased, and it coincided with EPS production. For that reason, we conducted additional experiments at different initial pH values to ascertain the role of pH in the production of EPS and potential causes for acidification. For a comprehensive understanding of the biopolymer production process and the pH maintenance mechanisms, we conducted a comparative bioinformatics analysis of publicly available data sets for *R. toruloides*, mapping the putative metabolic pathways involved in EPS biosynthesis and transport, as well as proton and cation balances. Together, our results show that the physiological pH decrease is caused by the utilization of inorganic nitrogen, and the resulting environment acidification triggers EPS production in *R. toruloides*. We expect our results to facilitate the elucidation of molecular mechanisms involved in the production of EPS in future studies, developing this yeast as an extracellular microbial biopolymer cell factory.

## MATERIALS AND METHODS

### Strain and culture conditions

The yeast *Rhodotorula toruloides* CCT0783 (synonym NBRC10076) (8, 37) is used in this study. The cultures were maintained in glycerol stocks, stored at −80°C, and aliquoted vials were thawed on ice for inoculation. Pre-culture cultivations were performed in a 50 mL Falcon tube containing 5 mL YPD medium and incubated overnight (200 rpm, 30°C) in a rotary shaker incubator (Excella E25, New Brunswick Scientific, USA). The obtained cells were then used as the inoculum in cultures with chemically defined media. YPD was thoroughly washed off the cells for the inoculation step by centrifuging (6,000 rpm, 20°C, 5 min) and resuspending them in the appropriate chemically defined media. A consistent inoculum size was achieved by measuring the pre-culture optical density at 600 nm (OD_600_) and ensuring the initial OD_600_ was 0.1 after inoculation in experiments. For the experiments starting at about pH 2 in chemically defined media, the initial OD_600_ was approximately 6.0. The cultivation experiments were performed using 250 mL sterile shaker flasks containing 25 mL of chemically defined media or YPD. The flasks were cultivated in a rotary incubator (200 rpm, 30°C) for 120 h. Samples for measuring dry cell weight, EPS, and pH values were collected as needed. All preparations and aliquoting were conducted under sterile conditions.

### Culture media: composition, preparation, and pH adjustment

YPD medium was used to revive cells from glycerol stocks and to prepare pre-cultures. One liter of YPD medium contained 20 g of peptone from meat (bacteriological grade, >95%, Thermo Fisher Scientific, USA), 10 g of yeast extract (Thermo Fisher Scientific, USA), and 22 g of D(+)-glucose monohydrate (>99.5%, Carl Roth GmbH, Germany). The YPD medium had its pH adjusted to 3 with a solution of HCl (1 mol/L) when needed.

The chemically defined culture media were prepared with Milli-Q water according to formulations described previously (8), with the following composition per liter: 3 g KH_2_PO_4_ (100%, Thermo Fisher Scientific, USA); 2 or 5 g (NH_4_)_2_SO_4_ (99.8%, Thermo Fisher Scientific, USA); 0.5 g MgSO_4_**·**7H_2_O (≥99%, Thermo Fisher Scientific, USA); 20 to 120 g D(+)-glucose monohydrate or ethanol (96% vol/vol). The pH was adjusted to 2 or 3 with an HCl solution (1 mol/L) or to 6 or 7 with an NaOH solution (2 mol/L) when needed in non-buffered pH-adjusted media. K_2_HPO_4_ (98%, 5.25 g/L) was added to give a pH of approximately 6.9 for the buffered cultivation media. KCl (≥99%, Lach-Ner S. R. O., Czech Republic) was added to the cultivation media in specific experiments to obtain a concentration of K^+^ ions equal to 0.1 mol/L. Vitamin and trace element solutions were added in the proportion of 1 mL per liter of medium. They were prepared and sterilized by filtration separately. The composition of the vitamin solution per liter of Milli-Q water (pH = 6.5) was biotin, 0.05 g; p-aminobenzoic acid, 0.2 g; nicotinic acid, 1 g; Ca-pantothenate, 1 g; pyridoxine-HCl, 1 g; thiamine-HCl, 1 g; and myoinositol (AppliChem GmbH, Germany), 25 g. The composition of the trace elements solution per liter of Milli-Q water was EDTA, 15 g; ZnSO_4_**·**7H_2_O, 4.5 g; MnCl_2_**·**2H_2_O, 0.84 g (or MnCl_2_**·**4H_2_O, 1.03 g); CoCl_2_**·**6H_2_O, 0.3 g; CuSO_4_**·**5H_2_O, 0.3 g; Na_2_MoO_4_**·**2H_2_O, 0.4 g; H_3_BO_3_, 1 g; KI, 0.1 g (8).

Thermal sterilization of the media components was conducted in an autoclave (Systec V-95, Systec, USA) at 121°C for 15 minutes. After the solutions were cooled to room temperature, they were mixed, and the vitamins and trace elements were added. All steps were performed in a sterile environment. The C:N ratios of different experimental conditions were varied by adjusting the initial concentrations of glucose and ethanol or ammonium sulfate and urea (equation 1), in which N_C_ and N_N_ represent the number of moles of carbon and nitrogen atoms per mole of the corresponding carbon and nitrogen sources; [C-source] and [N-source] represent the concentrations of the carbon and nitrogen sources (g/L), respectively; and MM_C_ and MM_N_ represent the molar masses (g/mol) of the carbon and nitrogen sources, respectively. The detailed concentrations of each compound to reach the desired C:N ratios are shown in Table S1.


(eq. 1) $$C\text{:}N\;=\;\left(\frac{N_{C}\;\times \;\left[C\text{-}source\right]}{MM_{C}}\right)÷\left(\frac{N_{N}\;\times \;\left[N\text{-}source\right]}{MM_{N}}\right)$$

### High-performance liquid chromatography (HPLC)

To obtain the initial concentrations of glucose and ethanol in the media, 1 mL of the medium was collected in 2 mL Eppendorf tubes, centrifuged (21,950 × *g*, 4°C, 5 min), and the supernatant was subsequently transferred to HPLC vials. The samples were filtered and diluted as necessary. Glucose and ethanol concentrations in the solution were quantified using an HPLC (LC-2030C Plus, Shimadzu, Kyoto, Japan) equipped with a refractive index detector (RID-20A, Shimadzu, Kyoto, Japan). The samples were stored at 4°C on the sample rack, and 20 µL aliquots were automatically injected into an Aminex HPX-87H ion exclusion column (Bio-Rad, USA). Elution with 5 mmol/L H_2_SO_4_ was performed in the isocratic mode at a flow rate of 0.6 mL/min with column and detector temperatures set to 45°C.

### pH measurements to monitor culture media acidification

To measure the initial pH value, 1 mL of the culture medium was transferred to a 2 mL Eppendorf tube, and the pH was determined using a pH meter (HACH HQ30d, Mettler Toledo, USA). For extra pH measurements at specified cultivation time points, the same volume was centrifuged (21,950 × *g*, 20°C, 5 min) to pellet the cells. Then, the supernatant was collected for pH measurement.

### Determination of dry cell weight

The dry cell weight of the biomass (DCW) was obtained by collecting 1 mL of the cultivation medium and then centrifuging it (21,950 × *g*, 5 min). The supernatant was removed, and 1 mL of PBS was added to resuspend the cells. The samples were vortexed, pipetted into pre-weighted 0.45 µm filter papers, washed with Milli-Q water, and filtered using a vacuum filtration system. The filter papers containing the biomass were dried in a microwave oven at 900 W for 2–3 minutes in 30 second intervals. The filter papers were stored in a desiccator until weighed the following day. The difference in the measured weights represented the DCW of the samples.

### Extracellular biopolymers: polysaccharides and proteins

#### Extraction and quantification

Following the procedures described in the literature (38, 39), the EPS were separated from the cell culture with minor modifications. Briefly, the culture medium was transferred to 50 mL Falcon tubes and centrifuged (6,000 × *g*, 4°C, 30 min). The supernatant was carefully separated from the cell pellet. To precipitate the EPS, cold ethanol (96% vol/vol, 4°C) was added to the supernatant at a ratio of 2 parts of ethanol to 1 part of supernatant in volume. The mixture was stored at 4°C for 24 h to promote EPS precipitation. After this period, a distinct layer of EPS formed at the middle or top of the liquid. This layer was carefully collected for further processing and analysis. Finally, the obtained EPS was placed in an incubator at 50°C until no change in mass was observed, indicating that all the excess liquid had evaporated. The obtained product was qualitatively confirmed to be a polysaccharide using the Alcian blue staining method with minor modifications (40). In brief, a stock solution was prepared by dissolving 1% wt/vol Alcian Blue 8 GX in 3% vol/vol acetic acid solution. The cell suspension was stained for 30 minutes in a diluted staining solution with a final concentration of 0.08% wt/vol Alcian Blue 8 GX. The stained cell suspension was observed under an Olympus Microscope CX21 (Olympus, Japan).

The quantification of protein by Bradford’s assay was performed with dry EPS samples (41) with Pierce Bradford Protein Assay Reagent (Thermo Fisher Scientific, USA), following the protocol for standard test tube procedures. Briefly, a known mass of dried EPS was dissolved in saline solution (0.9% wt/vol). Then, 30 µL of the samples or the standards was added to a cuvette containing 1.5 mL of the Bradford reagent. The cuvettes were incubated in the dark and at room temperature for 10 minutes. Then, the absorbance was measured at 595 nm. A bovine serum albumin (BSA) stock solution (2 mg/mL) was used as the standard to prepare the calibration curve following the supplier’s instructions.

#### Chemical characterizations

##### Fourier transform infrared (FTIR) spectroscopy analysis

For FTIR analysis, dry EPS, glucose, and dry biomass samples were carefully placed on the ATR crystal and analyzed on an FTIR spectrometer (IRTracer-100, Shimadzu, Kyoto, Japan) in the attenuated total reflection (ATR) mode. The spectra were recorded in the range of 4,000 to 400 cm⁻¹, with a spectral resolution of 6 cm⁻¹ and an aperture of 5 mm. The peaks identified by the software (LabSolutions, Shimadzu, Japan) were compared to a reference library to determine the functional groups present in the polysaccharide. The obtained spectra were processed for signal smoothing using the second derivative Savitzky-Golay method with 25 points in OriginPro version 2019b (OriginLab Corporation, USA) (42).

##### EPS composition

The monosaccharide composition of EPS was determined using methods adapted from Hamidi et al. (43). In brief, EPS (10 mg) was dissolved and hydrolyzed in 1 mL of trifluoroacetic acid (TFA, 2 mol/L) for 90 minutes in a dry bath at 120°C. The TFA was then evaporated under a nitrogen stream. One mL of sodium borohydride solution (NaBH_4_, 20 g/L) was added, and the reaction was maintained at room temperature for 3 h. The sample was dried again with nitrogen gas. For derivatization, the sample was treated with pyridine (300 µL) and acetic anhydride (400 µL), as described by Wang et al. (44). The resulting mixture was kept at room temperature (30 min) and heated to 90°C (30 min) under slow agitation. Then, the sample was centrifuged, and the supernatant was transferred to a new glass vial to separate the solids.

The clarified samples were transferred to a 7890A gas chromatograph coupled to a 5975 C mass spectrometer (Agilent Technologies, USA) with an electron ionization source and a quadrupole mass analyzer. One μL of the sample was injected in split mode (1:10) at 275°C. The flow rate of the carrier gas (helium) was 1.3 mL/min. The initial oven temperature was set to 50°C (1 min), increasing by 10°C/min until it reached 220°C (5 min), and increasing by 10°C/min until it reached 250°C (2 min). The total running time was 28 minutes. The analyte ionization was performed in the electron ionization mode with an electron energy of 70 eV. The interface, ion source, and mass analyzer temperatures were set at 280, 230, and 150°C, respectively. The scan mode in the target ion range, from 50 to 800 *m/z*, was employed to monitor the compounds of interest (43). Compounds were separated in a ZB-5plus capillary column (30 m x 0.25 mm x 0.25 µm, Agilent Technologies, USA). Agilent MassHunter Qualitative, Quantitative, and Unknown Analysis were used for data analyses. The retention times for different sugars were obtained with pure monosaccharide standards that underwent the same derivatization procedure.

### Comparative homology analysis of *R. toruloides* and mapping of putative EPS biosynthesis and transport pathways

In our experiments, we deployed the *R. toruloides* CCT0783 strain, which was previously sequenced and deposited, but without a functional annotation of the genome (5). Therefore, we performed a functional annotation of this genome sequence and compared it with *R. toruloides* IFO0880 (45), *R. toruloides* NP11 (46), and *Rhodotorula glutinis* ZHK (47) to identify homologies in genomic sequences, transcriptome expressions, and proteome relevant to the pathways investigated in this study.

#### Functional annotation of the *R. toruloides* CCT0783 genome

We deployed Braker3 (48–60), a fully automated genome annotation pipeline, to structurally annotate the genome of *R. toruloides* CCT0783, together with proteins of fungi compiled from the OrthoDB database (61), offering evolutionary and functional annotations of orthologous genes with a wider sampling of genomes. Braker3 was used with default parameters except for the addition of “—fungus” specifier. Reads were aligned to the CCT0783 genome using Hisat2 with a “—dta” parameter.

We utilized AnnoPRO (62) for functional annotation of GO terms. The thresholds for filtering the outputs were 0.25 for biological processes, 0.15 for molecular functions, and 0.29 for cellular components. The thresholds were chosen based on a test run using *Saccharomyces cerevisiae* S288C proteome and functional annotation from *yeastgenome.org* (63) to maximize the F1 score metric—a measure of the predictive performance of a model calculated based on precision and recall. CLEAN was used for the functional annotation of Enzyme Commission (EC) numbers. All EC numbers with confidence above 70% were added to the annotation (64). The annotated genome of *R. toruloides* strain CCT0783 was then used to identify sequence similarities to the closely related reference genomes, namely (i) *R. toruloides* strain IFO0880 reference genome v4.0 (Mycocosm database) (45) and (ii) *R. toruloides* strain NP11, taxid 1130832 (NCBI protein database) (46). Julia, a programming language, was used to combine the outputs of Braker3, AnnoPRO, CLEAN, and the homology to NP11 and IFO880 into a single annotation (65). Gene products were named based on NP11 names, IFO880 names, or names of corresponding EC numbers.

#### Sequence similarity analysis of the annotated genome

We further focused on sequence similarity analysis of genes relating to EPS biosynthesis and transport using NCBI *blastp* command line software to find matches in the CCT0783 proteome for EPS and transporter genes from reference strains NP11 and IFO880, which, in turn, were obtained from UniProt (66).

#### Mapping the EPS biosynthesis and transport pathways and the regulators of pH maintenance

A previous experimental study using *R. glutinis* ZHK yeast reported the presence of an EPS biosynthesis pathway and potential transporters for EPS export (47). However, similar information is lacking for other *R. toruloides* strains, including CCT0783. For this reason, we performed the pathway mapping using the *R. glutinis* ZHK genomic data set, which was retrieved from the NCBI (genome assembly ASM1550198v1) (67) and queried in a blast search against the reference genomes of *R. toruloides* IFO0880 and NP11 strains (45, 46). The default parameters (E = 1e5, Filter = True, BLOSUM62) were used for the similarity analysis.

To complement the mentioned analysis and gain a system-level view of pathways, we queried genome-scale models of *R. toruloides*. These genome-scale models are based on genomic information from *R. toruloides* reference genomes and are used to stoichiometrically reconstruct enzymatic and transport reactions through gene-protein relation (GPR) information, which allowed us to query the genes corresponding to biochemical reactions in reference genomes. Genome-scale models of *R. toruloides* have been reconstructed using models of *Saccharomyces cerevisiae*, *Chlamydomonas reinhardtii*, human, mouse, *Escherichia coli*, and *Pseudomonas putida*, covering most of the orthologous proteins in *R. toruloides* (68). We specifically queried the implicated pathway information from *R. glutinis* ZHK (47) in the genome-scale models of IFO0880 and NP11 strains to identify potential genes involved in the synthesis of nucleoside diphosphate sugars (NDP sugars): “glucose 6-phosphate” (g6p), “glucose 1-phosphate” (g1p), “mannose 6-phosphate” (man6p), “mannose 1-phosphate” (Man1P), “UDP-glucose” (UDP-glu), “UDP-galactose” (UDP-gal), and “GDP-mannose” (GDP-man) reactions (68, 69). Additionally, we utilized available information on EPS biosynthesis in other organisms, wherein gene candidates of glycosyl and mannosyl transferases are implicated in EPS production. Therefore, we searched publicly available omics data sets for *R. toruloides* strains using the keywords “glycosyltransferase” and “mannosyltransferase” (7, 68), which allowed us to obtain the corresponding genes from the reference genomes.

Subsequently, we considered potential EPS transport candidates based on previous studies in other yeasts (8). Here, we mapped two types of transporters: (i) within cell organelles, such as nucleotide-sugar transporters (NSTs) and intracellular proton transporters, and (ii) transporters on the cell membrane leading to the export of biopolymers. The transporters were searched from publicly available omics data sets using the keywords “UDP-,” “sugar,” “triose-phosphate,” “transporter,” “SLC,” “antiporter,” “ATPase,” and “synthase” (7, 68). All the above allowed us to map genes in reference genomes, which were then used for sequence similarity analysis in the CCT0783 strain.

Finally, since several omics studies have been published on *R. toruloides* reference strains, including growth conditions, we used these data to compare the genes with high sequence similarity to the CCT0783 strain and to ascertain the presence of protein expression levels from relevant available omics data sets. Moreover, to better understand the synergy between nitrogen assimilation and the acidification of the CCT0783 culture, we also verified the presence of nitrogen transporters and ion exchangers that may be involved in maintaining intracellular pH and biopolymer production in this yeast.

## RESULTS

### Characterizing *R. toruloides* exopolysaccharides

**Fig 1:**
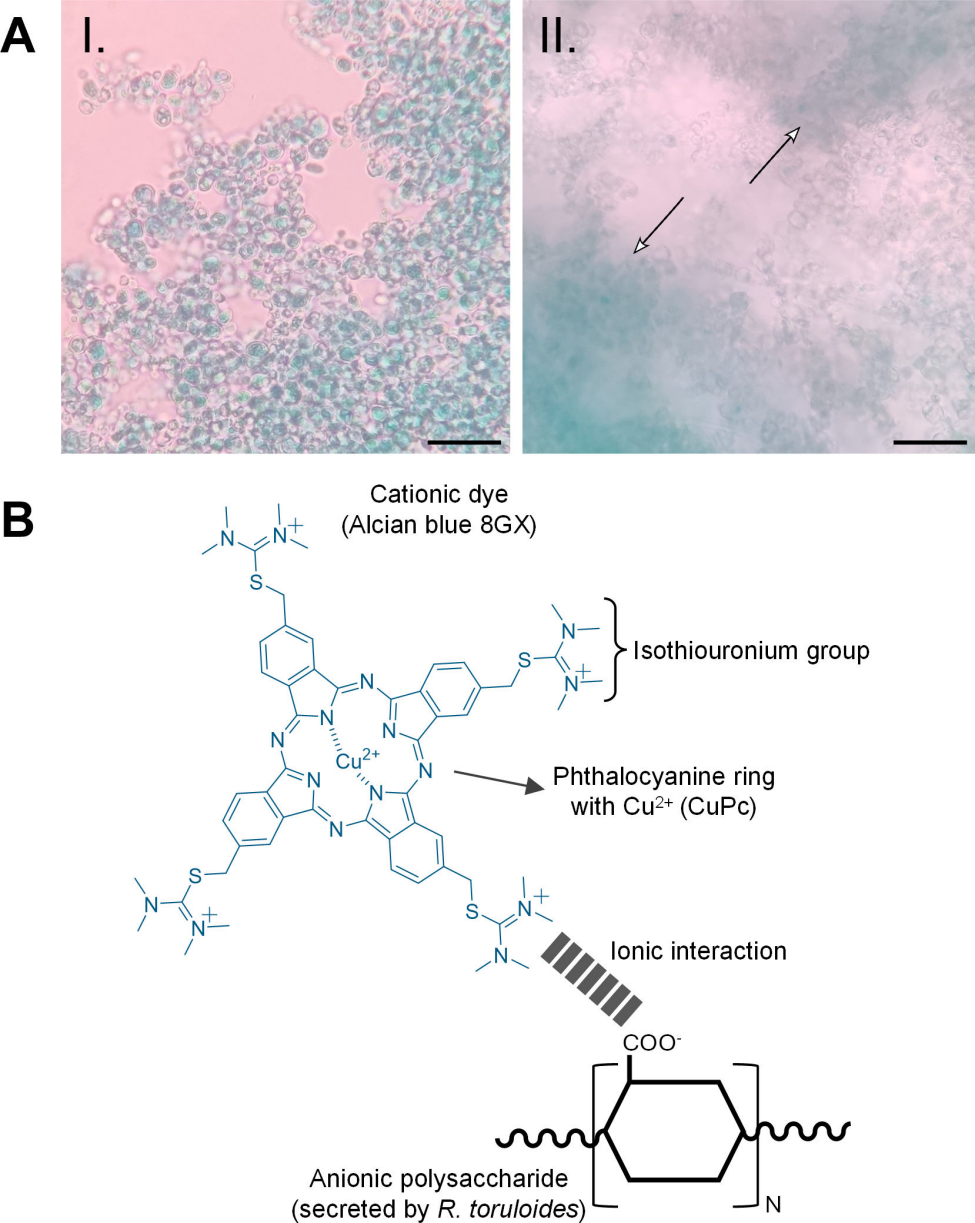
Qualitative identification of *R. toruloides’* exopolysaccharides. (**A**) EPS staining with alcian blue before (I) and after (II) precipitation with cold ethanol 96% vol/vol. Arrows point to precipitated EPS. Scale bar = 50 µm; (**B**) Alcian blue stain interaction mechanism with anionic polysaccharides involves ionic interactions between charged groups.

Our previous study of *R. toruloides* CCT0783 suspension cultures showed cellular aggregation in the glucose-containing chemically defined medium (8). In the study, we found that supplementing KCl or NaCl (0.1 to 1.0 mol/L) inhibited cellular aggregate formation. However, the nature of extracellular biomolecules involved in the development of cellular aggregates remained unexplored in the previous study. Hence, we qualitatively and quantitatively identified and characterized extracellular macromolecules relating to aggregate formation. First, we examined whether carbohydrate EPS was present in aggregate-inducing conditions since extracellular matrices in biofilms and microbial aggregates reportedly contain carbohydrates, proteins, and other macromolecules, depending on the nutrient environment (70, 71). For this, we deployed a qualitative method using the Alcian blue stain (40). This cationic dye binds to anionic polysaccharides through ionic interactions between its tetramethylisothiouronium (-SC(N(CH_3_)_2_)_2_^+^) groups and deprotonated carboxylate (-COO^−^) or sulfate (-OSO_3_^−^) groups of the polysaccharides at low pH (72). The stained samples, obtained by ethanol precipitation, showed a blue and cloudy matrix, indicating it is a carbohydrate-rich polymer (Fig. 1A). In this staining process, the phthalocyanine ring with a copper ion gives blue color to the reaction product (Fig. 1B).

**Fig 2:**
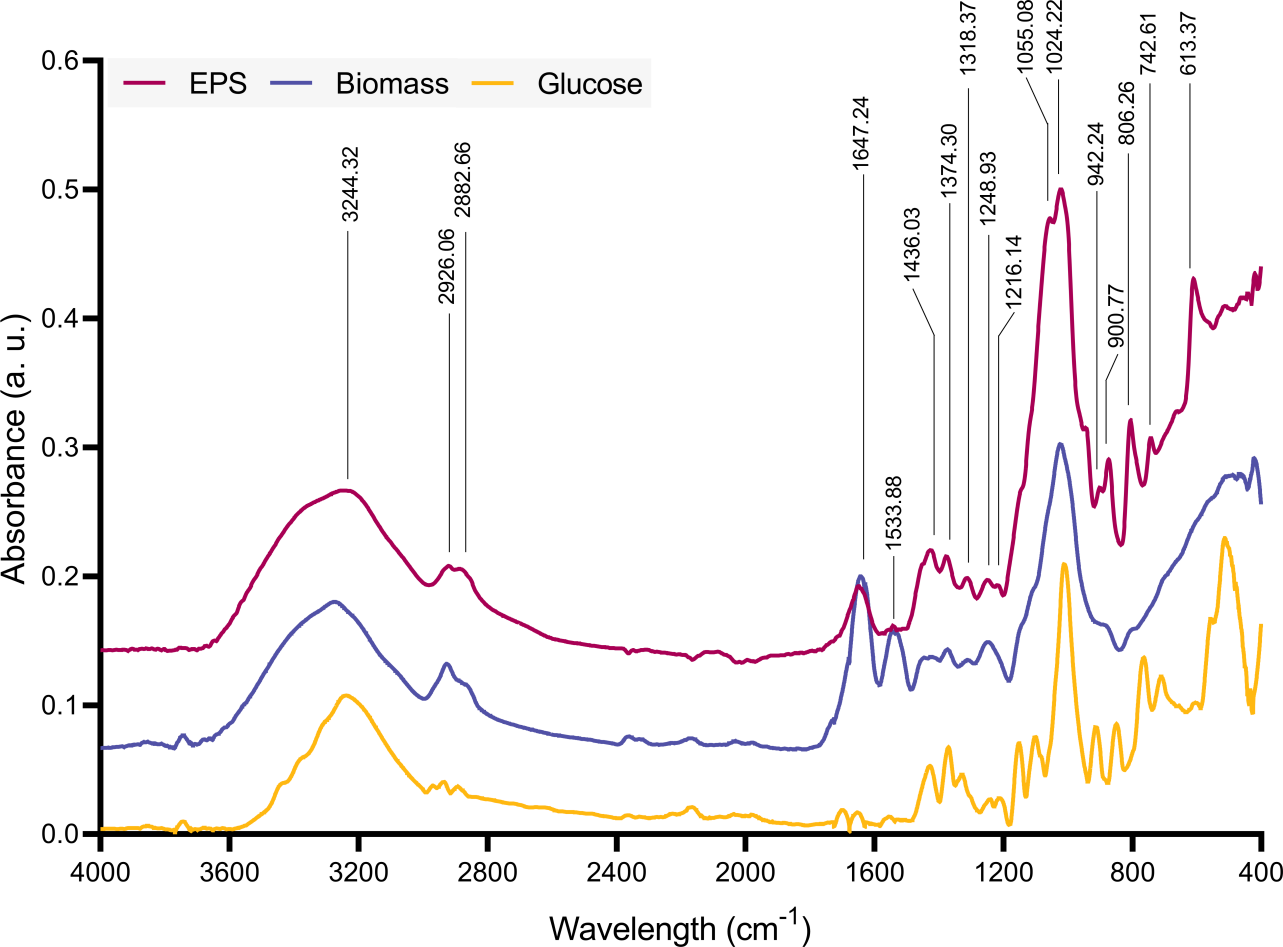
Representative FTIR spectra of dry EPS (red), dry biomass (blue), and glucose (yellow).

Second, we ascertained the chemical fingerprints of the potential biopolymers in the crude extracts from *R. toruloides* cultures. To achieve this, we performed FTIR analysis on the dry EPS samples to determine the functional groups in the material. The FTIR results showed characteristic peaks of carbohydrate polymers (Fig. 2). The most significant peaks are presented in Table 1. The spectrum exhibits a broad peak for O-H stretching at 3,244 cm^−1^. A double peak for aliphatic C-H stretching was observed at 2,926 and 2,882 cm^−1^. The peak at 1,647 cm^−1^ corresponds to -O-H bending from water strongly bound to the EPS and/or C = O stretching. Combined with peaks at 1,436 and 1,374 cm^−1^, the EPS is likely acidic (34, 43). The ether peak for -CH_2_-O-CH_2_- was identified by two strong bands at 1,055 and 1,024 cm^−1^, usually between 1,200 and 1,000 cm^−1^. Peaks from 950 to 800 cm^−1^ indicate the presence of both α- and β-glycosidic bonds (73). The peaks in the 1,400 to 1,200 cm^−1^ region could be attributed to impurities, including proteins and lipids in the crude EPS sample. To identify the material as a polysaccharide, dry biomass and glucose spectra were obtained for comparison (Fig. 2). These spectra show matching peaks to the EPS spectrum, including peaks related to O-H stretching and aliphatic C-H. However, the double peak related to ether groups and glycosidic bonds is missing. Interestingly, the biomass spectrum exhibits a unique peak in the 1,580–1,520 region, which is associated with the bending in -CONH groups (74). Table S2 summarizes the FTIR data from EPS derived from other *Rhodotorula* yeasts reported in the literature, with matching peaks at approximately 3,400 to 3,200 cm^−1^, 2,930 cm^−1^, 1,640 cm^−1^, and from 1,200 to 1,000 cm^−1^, confirming that our extracted material is mainly composed of a polysaccharide.

**TABLE 1:** FTIR peak assignments for oven-dried EPS samples

Peaks (cm^−1^)	Assignments
3,244	O-H stretching vibration
2,926, 2,882	Aliphatic C-H stretching vibration
1,647	-O-H (H_2_O) bending vibration and/or C = O (carbonyl) asymmetrical stretching vibration
1,436, 1,374	C = O (carboxyl) symmetric stretching vibration
1,400–1,200	Impurities from proteins, lipids, and nucleic acids
1,200–1,000	C-O and CH_2_-O-CH_2_ stretching vibration
950–850	α- and β-glycosidic bond stretching vibration

**Fig 3:**
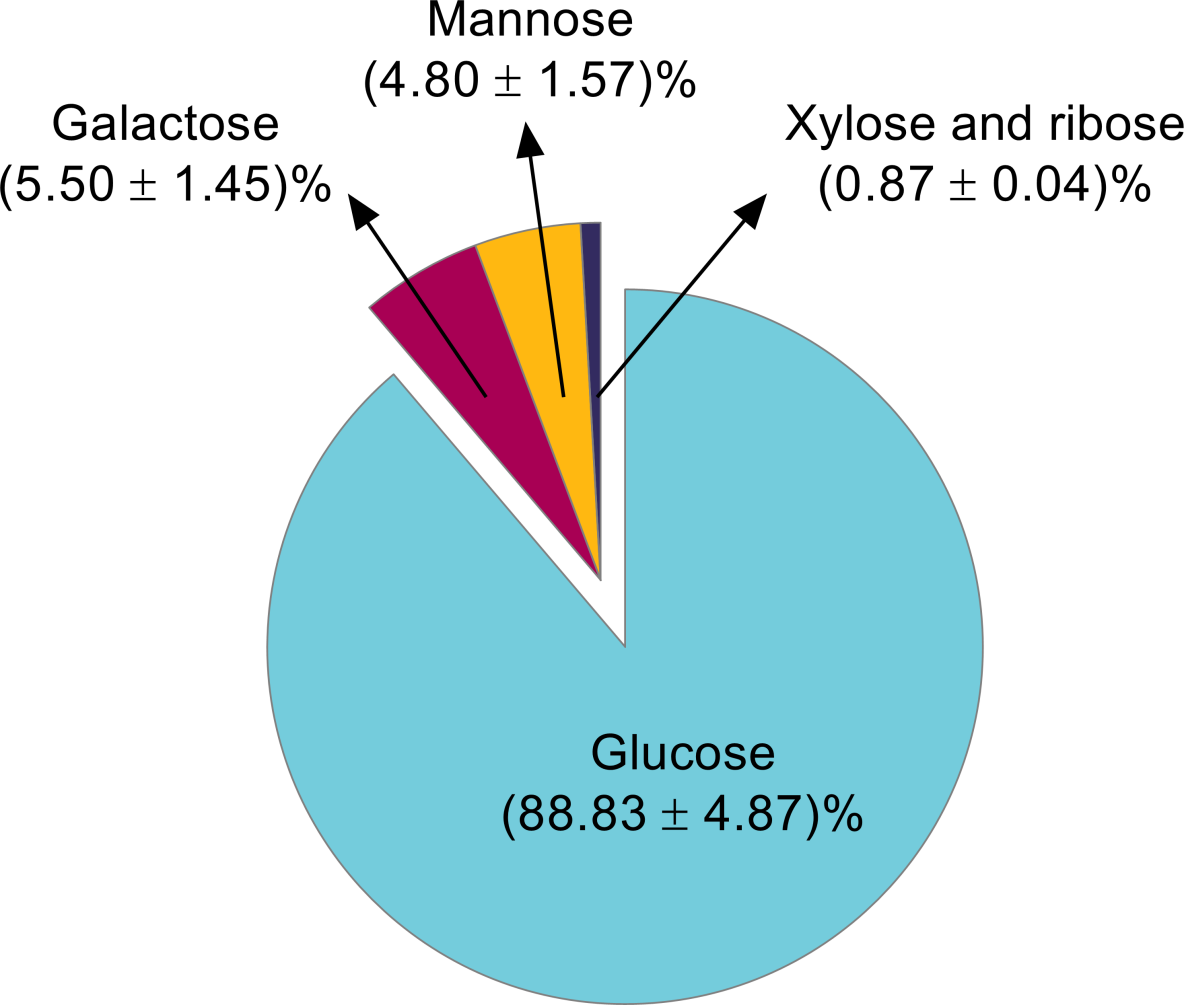
Monosaccharide composition of the carbohydrate fraction of EPS obtained through GC-MS analysis. The standard deviation corresponds to three independent experiments.

The determination of the repeating units in the biopolymer was conducted by hydrolyzing the EPS using a TFA solution (2 mol/L), reducing the product with an NaBH_4_ solution (20 g/L), and acetylating it in a mixture of pyridine and acetic anhydride. GC-MS analysis of the treated EPS revealed that the monosaccharide composition of the carbohydrate fraction consisted of glucose (88.83 ± 4.87%), galactose (5.50 ± 1.50%), and mannose (4.80 ± 1.57%). Xylose and ribose were present in smaller quantities, accounting for a total of 0.87 ± 0.04% of the carbohydrates (Fig. 3). Although the EPS was evaluated as possibly negatively charged by the Alcian blue staining method and by acidic groups in the FTIR spectra, their detection could have been hindered by the difficulty in obtaining their derived products in the method applied for hydrolysis and derivation in this study (44, 75). The Bradford assay, using bovine serum albumin as the standard, indicated the presence of proteins. The assayed samples contained a protein fraction ranging from 0.8% to 1.0% of their dry weight.

### Evaluating EPS biosynthesis conditions

**Fig 4:**
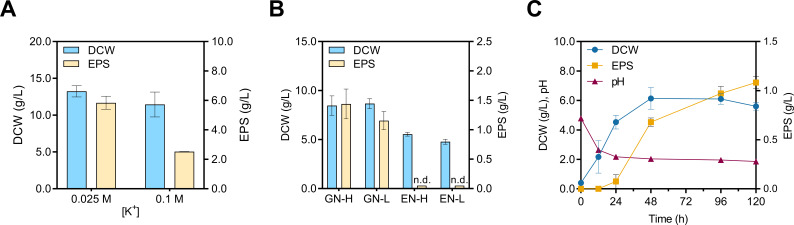
*R. toruloides* physiology in response to KCl, carbon, and nitrogen changes in culture media. (**A**) Effect of KCl supplementation on biomass and EPS production. DCW and EPS (g/L) in chemically defined medium with 120 g/L of glucose and 5 g/L of (NH_4_)_2_SO_4_ (C:N ratio = 52.8) supplemented with 0.1 M of KCl, compared to no supplementation, to increase [K^+^]. (**B**) Variation in the carbon source (glucose or ethanol) at 20 g/L and nitrogen source concentration at 2.0 or 5.0 g/L (C:N ratios: GN-H = 8.8; GN-L = 22.0; EN-H = 11.5; EN-L = 28.7) for dry cell weight (DCW) and exopolysaccharide (EPS) production quantification. n.d. means no EPS was detected during quantification. (**C**) An example time-series experiment showing DCW (g/L), pH, and EPS (g/L) graphs for cultivation up to 120 h in GN-L. The standard deviation corresponds to three independent experiments.

Having identified and characterized the EPS in conditions that enable cellular aggregation, we asked whether EPS is also produced irrespective of aggregate formation. We indeed found that *R. toruloides* CCT0783 produces EPS not only when cells form aggregates (Fig. 4). We did this by assessing the role of potassium salts in the EPS production, which we previously reported in qualitative observation as prohibiting cellular aggregate formation in the suspension cultures (8). Our results confirmed the previous study that supplementing cultures with additional potassium salts (KCl, 0.1 mol/L) caused them to remain free of cellular aggregates, without a significant reduction in DCW (Fig. 4A), decreasing from 13.23 ± 0.76 to 11.46 ± 1.69 g/L (C:N ratio = 52.8). However, we found that a higher ionic strength reduces EPS production by more than 50% in *R. toruloides* cultures, which likely contributes to the reduction in cellular aggregates.

Subsequently, we investigated the potential metabolic route of biopolymer production in *R. toruloides*. For this, we cultured *R. toruloides* in glucose or ethanol-rich medium (Fig. 4B). We found that cultures grown in ethanol, a gluconeogenic carbon source, did not produce any detectable EPS, despite growth. Moreover, the results showed that the C:N ratio in the culture media influenced EPS production. At 5 g/L of ammonium sulfate, an EPS titer of 1.44 ± 0.25 g/L was obtained (GN-H, C:N ratio = 8.8), while the titer of EPS was equal to 1.16 ± 0.15 g/L for 2 g/L of ammonium sulfate (GN-L, C:N ratio = 22.0) (Fig. 4B). In these experiments, the culture media initial pH was not fixed to a specific value and ranged from 4.40 to 4.57. We observed that aggregate formation occurs during the early log phase for the conditions containing glucose with different nitrogen concentrations and dissipates after 24 to 48 h. We did not observe any cellular aggregation for ethanol-grown cultures. These results suggest that the glycolysis pathway provides the carbon backbones to produce EPS.

We obtained the temporal profile of EPS production over a 120 h cultivation period for one of the EPS production conditions (GN-L, C:N ratio = 22.0) (Fig. 4C). We observed that the EPS production occurs mostly during the transition from the growth phase to the stationary phase, reaching 1.08 ± 0.06 g/L after 120 h of cultivation. The biomass formation was associated with the acidification of the medium, from 4.79 to 2.18, during the first 24 h, during which only a fraction of EPS was produced. As the culture transitioned to the stationary phase, a further decrease in the pH from 2.18 to 1.86 occurred, which corresponded with most of the EPS production (Fig. 4C). Therefore, for subsequent experiments focused on understanding the physiological conditions associated with the final EPS titers, glucose was added as the sole carbon source, and EPS extraction was performed after 120 h of cultivation.

### Investigating the contributions of C:N ratio, nitrogen sources, and pH in EPS production

**Fig 5:**
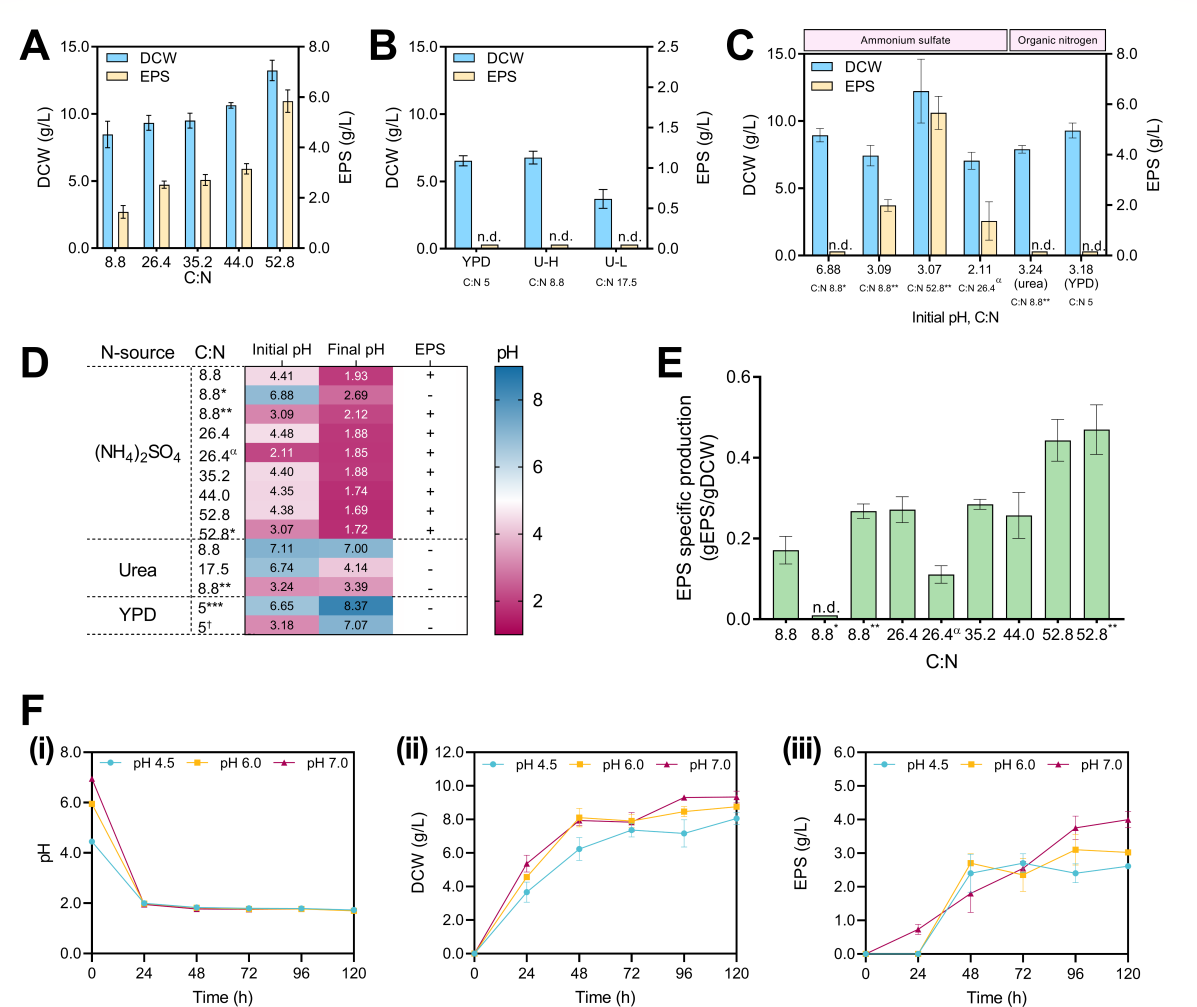
Evaluation of *R. toruloides* cultures in various culture media and cultivation conditions. (**A**) DCW (g/L) and EPS (g/L) production at various C:N ratios (8.8, 26.4, 35.2, 44.0, and 52.8); (NH_4_)_2_SO_4_ concentration was constant at 5 g/L; glucose concentrations were 20, 60, 80, 100, and 120 g/L, respectively. (**B**) DCW (g/L) and EPS (g/L) in nutrient-rich medium (YPD) and chemically defined media with C:N ratios 8.8 (U–H) and 17.5 (U–L), the glucose concentration was 20 g/L. (**C**) DCW (g/L) and EPS (g/L) in media with different C:N ratios, nitrogen sources, and starting pH levels. Concentrations of carbon and nitrogen sources are shown in Table S1. (**D**) Initial and final (after 120 h of cultivation) pH values, alongside the presence or absence of EPS in samples in different cultivation media separated by the nitrogen source and C:N ratio. Data were separated by nitrogen source as described in A, B, and C. (**E**) EPS specific production on biomass (gEPS/gDCW) after 120 h of cultivation in chemically defined media for different C:N ratios with (NH_4_)_2_SO_4_ as the nitrogen source. Abbreviations: (*) phosphate-buffered condition with initial pH equal to 6.88. (**) Initial pH adjusted to 3 with HCl (1 mol/L) with C:N ratios 8.8 and 52.4. (α) Initial OD_600_ = 6.0 and initial pH adjusted to 2 with HCl (1 mol/L). (***) Estimated C:N ratio for YPD media is 5. (†) Initial pH adjusted to 3 in YPD media. (+) and (−) indicate EPS was detected or not detected, respectively. n.d. means no EPS was detected. (**F**) (i) pH; (ii) DCW (g/L); (iii) EPS (g/L) over 120 h obtained by cultivating the yeast in different initial pH values (4.5, 6.0, and 7.0) at C:N ratio = 26.4 (glucose: 60 g/L; (NH_4_)_2_SO_4_: 5 g/L). Only initial glucose concentrations were quantified. The standard deviation corresponds to three independent experiments.

After observing the role of glycolysis in EPS production, we asked whether different C:N ratios in the culture media would affect EPS production. To achieve this, glucose concentration was increased from 20 to 120 g/L, and ammonium sulfate concentration was kept constant at 5 g/L in different triplicate batch culture experiments, giving C:N ratios from 8.8 to 52.8 (Fig. 5A). We observed that the increased supply of glucose in distinct media caused an increase in DCW (8.47 ± 0.99 to 13.23 ± 0.76 g/L) and in produced EPS (1.44 ± 0.25 to 5.84 ± 0.45 g/L).

Further, since ammonium salt concentrations in our preliminary experiments appeared to influence EPS production (Fig. 4B), we examined whether organic nitrogen sources, such as urea (C:N ratios 8.8 and 17.5) in chemically defined media or a nutrient-rich YPD medium (approximate C:N ratio = 5), where amino acids in yeast extract and peptone provide the nitrogen supply, would affect the EPS production. Under these conditions, the yeast grew to reach DCWs of 6.53 ± 0.37 g/L in the YPD medium, and 3.70 ± 0.70 and 6.77 ± 0.47 g/L for U-L (C:N ratio 17.5) and U-H (C:N ratio 8.8), respectively. Interestingly, no EPS was detected (n.d.) in any of the organic nitrogen conditions investigated (Fig. 5B).

Our results suggest that nitrogen metabolism plays a role in influencing EPS biosynthesis (Fig. 5A and B). Previous studies have shown that inorganic and organic nitrogen source uptake in yeasts involves distinct pH maintenance mechanisms (31, 76). Considering this, we investigated EPS production at different pH levels using both buffered and non-buffered media, and with inorganic or organic nitrogen sources (Fig. 5C). For the inorganic nitrogen conditions, we started by quantifying DCW and EPS from a buffered medium with an initial pH of 6.88 and from a non-buffered medium acidified to 3.09 with 1 mol/L HCl, while maintaining the C:N ratio of 8.8 in both cases. The biomass and EPS formation differed between the buffered (DCW: 8.95 ± 0.49 g/L, EPS: not detected) and non-buffered cultivations (DCW: 7.43 ± 0.76 g/L, EPS: 1.99 ± 0.23 g/L). When compared to the non-adjusted pH condition (pH approximately 4.5), a lower starting pH reduced the biomass by 12% (*P*-value < 0.1) and increased the EPS by 38% (*P*-value < 0.05) (Fig. 5A and C). For the condition with a C:N ratio of 52.8 and an initial pH of 3.07, we observed DCW and EPS values of 12.23 ± 2.37 and 5.66 ± 0.65 g/L, respectively, which were not significantly different (*P*-values > 0.5) from the results obtained under non-adjusted pH conditions (Fig. 5A and C). Next, we adjusted the initial pH of the media to 2.11 (C:N ratio 26.4) to check if EPS production would still occur at this pH value. The starting OD_600_ of 0.1 under these conditions did not allow cell growth (data not shown). Therefore, we repeated the experiment but with a starting OD_600_ of 6.0 (DCW: 5.43 ± 0.68 g/L). In this state, cell growth and EPS production were both detected after 120 h (final DCW: 7.15 ± 0.78 g/L, final EPS: 1.37 ± 0.76 g/L), while the pH decreased to 1.85 (Fig. 5C). The results suggest a complex interplay between the C:N ratio and initial pH for cell growth, physiological pH drop, and EPS production. For the organic nitrogen sources, we tested pH-adjusted YPD (initial pH 3.18) and urea (initial pH 3.24) media with C:N ratio equal to 5 and 8.8, respectively, to determine if a starting low pH would trigger EPS production; however, EPS was not detected in any of these conditions (Fig. 5C). Together, our data imply that inorganic nitrogen metabolism, which is directly connected to extracellular acidification of the culture media (76), is relevant to EPS production in *R. toruloides*.

To investigate a putative association between EPS synthesis and pH, we measured the initial and final pH values across all tested cultivation conditions (Fig. 5D). The media supplemented with ammonium sulfate, non-buffered conditions, and non-adjusted pH conditions had initial pH ranging from 4.35 to 4.48, which after 120 h of cultivation decreased to values at or below 2 in all tested conditions, and EPS was detected. Our findings indicate an apparent pH threshold for EPS synthesis under inorganic nitrogen-containing cultures. For the media with organic nitrogen, whether pH was adjusted or not, EPS was not detected. In urea-containing media, the final pH appeared to be dependent on the urea availability. The pH of the YPD media increased after 120 h, from 6.65 to 8.37, and from 3.18 to 7.17 in the pH-adjusted condition. The pH increase in YPD media is likely due to the consumption of amino acids present in the rich medium, which is consistent with previous studies (77).

The specific production of EPS on biomass (gEPS/gDCW) was calculated for the cultures supplemented with ammonium sulfate (Fig. 5E). Among the conditions with a C:N ratio of 8.8, the culture with an initial pH of 3.09 produced 0.27 ± 0.02 gEPS/gDCW, whereas the condition starting at pH 6.88 did not produce detectable EPS. At higher C:N ratios of 26.4, 35.2, and 44.0, the specific EPS production values were 0.27 ± 0.03, 0.28 ± 0.01, and 0.26 ± 0.06 gEPS/gDCW, respectively. When starting at pH 2 and higher OD_600_, the C:N ratio of 26.4 had a specific production of 0.11 ± 0.02 gEPS/gDCW. Finally, a maximum of 0.44 ± 0.05 gEPS/gDCW was observed at a C:N ratio of 52.8. When the same medium was acidified to an initial pH of 3, the specific EPS production did not significantly increase (0.48 ± 0.06 gEPS/gDCW, *P*-value > 0.5).

To further validate our results regarding the apparent pH threshold required for EPS production, we adjusted the pH of the media (C:N ratio 26.4) to 6.0 and 7.0 with NaOH (2 mol/L) and compared the cultivation results with the control condition (pH 4.5) for pH, DCW, and EPS over time (Fig. 5F). Our results show that for all conditions, the pH dropped to 2 after 24 h of cultivation and slightly reduced to 1.70–1.80 over 120 h, regardless of the initial pH value as the tested media are non-buffered (Fig. 5F-i). The cell growth, evaluated through the DCW, showed a similar profile for all tested conditions, rapidly increasing in the first 48 hours and then stabilizing. The final DCWs reveal a potential influence of pH on cell growth, where a lower pH caused a decrease in final DCW. For the pH values of 4.5, 6.0, and 7.0, the obtained DCW was 8.07 ± 0.25 g/L, 8.77 ± 0.06 g/L, and 9.33 ± 0.35 g/L, respectively (Fig. 5F-ii). EPS production (g/L) followed a similar production profile to DCW, in which a higher starting pH caused an increase in EPS production. Moreover, for the conditions at pH 4.5 and 6.0, EPS was first detected at 48 h of cultivation and remained virtually unchanged until the end of cultivation. The concentration of EPS in the cultures was 2.61 ± 0.23 g/L and 3.02 ± 0.46 g/L for the initial pH values of 4.5 and 6.0, respectively. In contrast, the cultivations starting at pH 7 had a relatively linear EPS production until 96 h of cultivation, reaching a maximum value at 120 h (4.00 ± 0.24 g/L), which corresponds to a 53% increase in EPS compared to the condition with non-adjusted pH (Fig. 5F-iii).

### Comparative analysis of *R. toruloides* strains, focusing on a putative EPS biosynthesis pathway

To understand the potential metabolic pathways involved in the biosynthesis and extracellular biopolymer production in *R. toruloides,* we utilized available genomic, transcriptomic, and proteomic data sets. These data sets allowed us to perform a comparative analysis of *R. toruloides* strains, whose identifiable attributes were mapped for the CCT0783 strain, making its genome more accessible for further investigations. We retrieved the genome sequence of *R. toruloides* CCT0783 from NCBI (5) and performed a functional annotation of this genome (PRJNA1231390).

**Fig 6:**
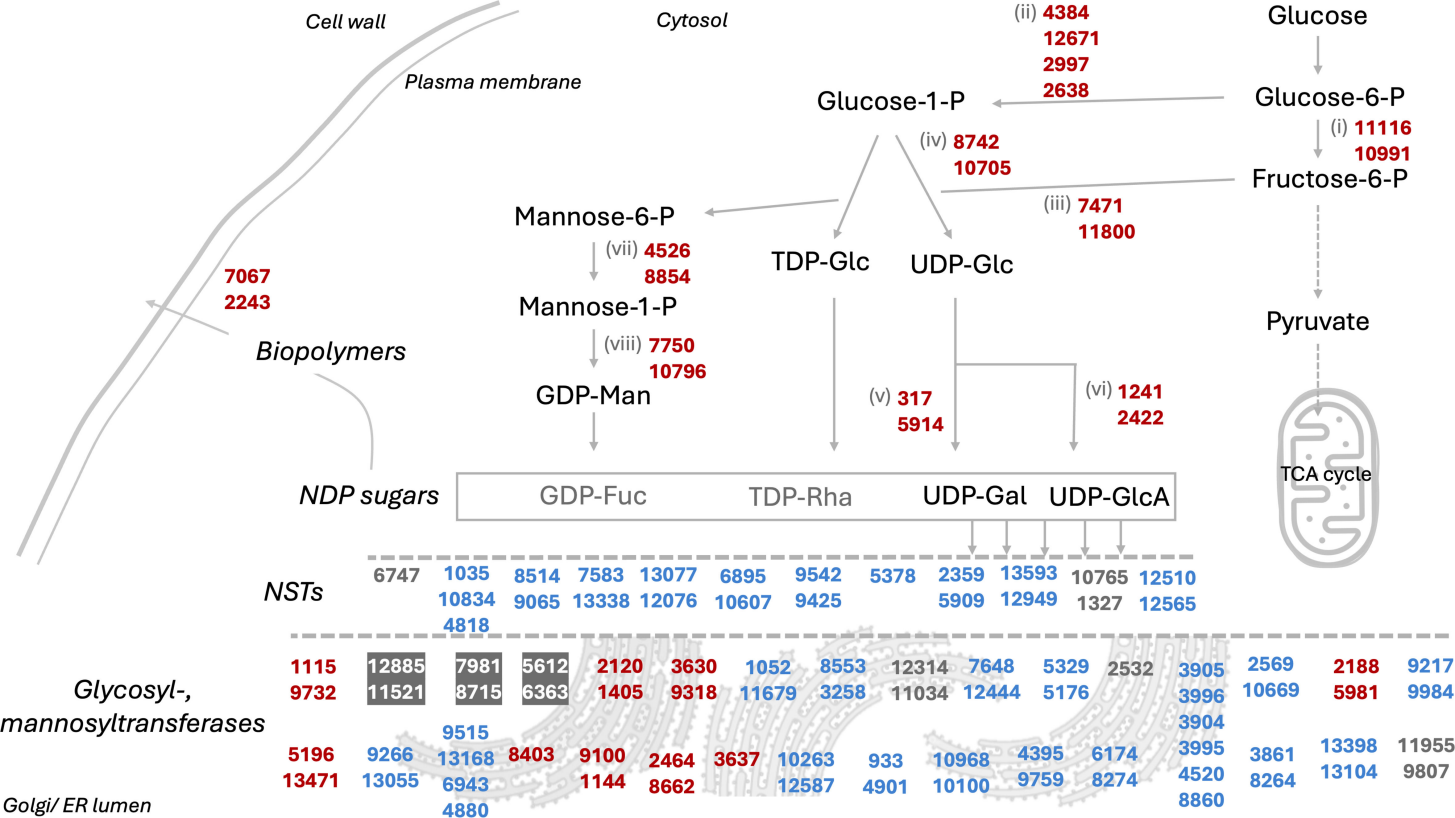
Proposed EPS biosynthesis and transport pathway in *R. toruloides* CCT0783, based on its genome annotation and representative gene expression data from previous omics studies. Names of genes, including reference studies, are listed in Tables S4 to S7. The mapped genes’ encoded proteins are visualized based on previous proteomics studies, highlighting differences in relative protein detection under EPS-inducing conditions: higher detection (red), lower detection (blue), and undetected proteins (gray). Proteins detected under control conditions are represented with a gray background. Abbreviations: (i) glucose-6-phosphate isomerase; (ii) phosphoglucomutase; (iii) mannose-6-phosphate isomerase; (iv) UTP-glucose-1-phosphate uridylyltransferase; (v) UDP-glucose-4-epimerase; (vi) UDP-glucose-6-dehydrogenase; (vii) phosphomannomutase; (viii) mannose-1-phosphate guanylyltransferase.

We used the annotated genome and data from previous studies to map and identify corresponding EPS biosynthesis and transport pathways. For this, we used available *Rhodotorula glutinis* ZHK experimental data that suggested an EPS biosynthesis pathway in this yeast (47). Moreover, we categorized the existing *Rhodotorula* omics studies into two categories: potentially EPS-inducing conditions, where pH remained uncontrolled, and EPS-non-inducing conditions, where pH was controlled during cultivation (Table S3). In the absence of direct omics studies for the CCT0783 strain, this categorization enabled us to use homology and similarity searches to infer relative proteome changes in the putative EPS biosynthesis and transport pathways in EPS-producing and non-producing conditions (Table S3; Fig. 6).

In a comparative gene sequence homology analysis with the reference *R. toruloides* strains (NP11 and IFO0880), CCT0783 strain genes encoding for NDP sugars (activated sugar donors for glycosylation reactions), NSTs (transport NDP sugars into the lumen of the Golgi or ER), glycosyl- and mannosyl transferases (catalyze the transfer of sugar residues from NDP sugars to growing polysaccharide chains), which are likely involved in the biosynthesis of EPS, showed 83% to 100% nucleotide similarity (Table S4; Fig. 6). We used this pathway sequence similarity information to estimate relative changes in the protein expressions for the EPS-producing and non-producing conditions (Table S4; Fig. 6). In the proteomics data, enzymes synthesizing NDP sugars are highly abundant in *R. toruloides*; however, neither upregulation nor downregulation on the protein level was observed by Kim et al. (68) during EPS-inducing conditions (Table S5). In a comparison of exponential and stationary growth phases, Zhao and Li reported several NSTs upregulated in EPS-induced conditions in *R. glutinis* ZHK (47). Therefore, to identify these genes in other *R. toruloides* strains, the gene sequences from *R. glutinis* ZHK were first retrieved from NCBI using annotations from Li et al. (67) and then blasted against the genome of the reference *R. toruloides* strains. These Blast results identified several candidate genes with an identity score above 77% (Table S6). However, most NSTs were not identified or had very low abundance in the existing proteomics studies that used EPS-inducing conditions (Tables S6 and S7). This search also identified the glycosyltransferase RHTO_00642 with an identity score of 77.10% (Table S6), which was found to be abundant but downregulated under EPS-inducing conditions (Table S7). We identified several SLC35 genes in *R. toruloides* genomes, which encode a protein family responsible for transporting NSTs from the cytosol or nucleus to the ER or Golgi lumen (78) (Table S7). The proteins encoded by those genes were identified as low abundant in *R. toruloides* under EPS-inducing conditions by Kim and colleagues (68) (Table S7). However, some of them were not identified in the genome of CCT0783. NSTs are closely related to triose phosphate translocators (TPT) genes (79), and we identified a few TPTs in low concentrations in EPS-inducing conditions (Table S7). Many mannosyltransferases responsible for glycosyl phosphatidylinositol synthesis in yeast are also present in *R. toruloides* (80); some are highly abundant at the proteome level (Table S7). Interestingly, the most abundant mannosyltransferases are downregulated during EPS production conditions but upregulated during the categorized control conditions. Based on the functional annotation of CCT0783 and a comparative homology analysis of available data, we propose a general pathway for the EPS biosynthesis in *R. toruloides* (Fig. 6). Though beyond the scope of the present study, we envision that a future experimental validation and analysis of the proposed pathway using the direct omics data sets for the biopolymers’ producing and non-producing conditions would be valuable.

### Unraveling the role of pH in biopolymer production

**Fig 7:**
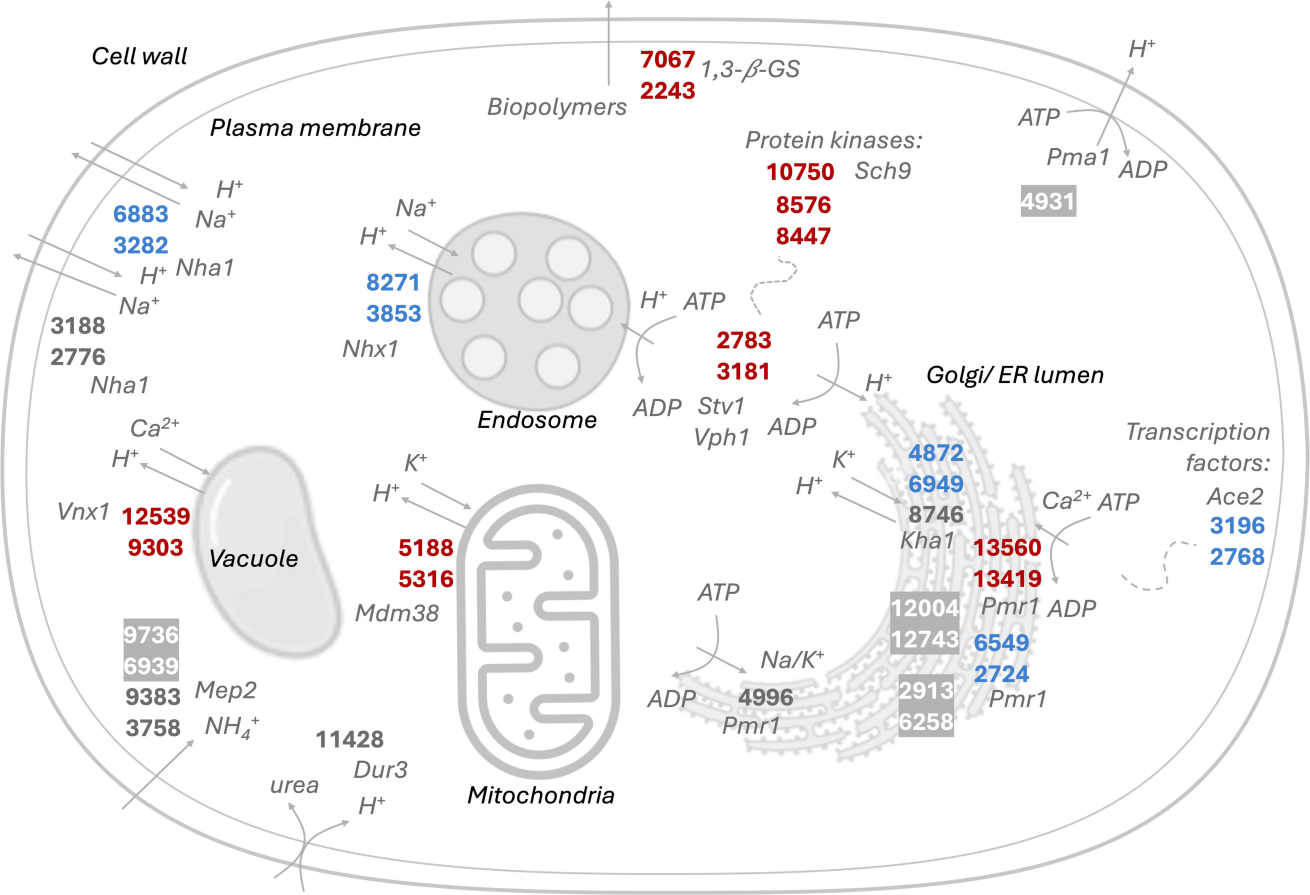
Putative proton, nitrogen, and biopolymer transporters in *R. toruloides* CCT0783, based on its genome annotation and representative protein expression data from previous omics studies. Names of genes are listed in Tables S8 and S9. The mapped genes’ encoded proteins are visualized based on previous proteomics studies, highlighting differences in relative protein detection under EPS-inducing conditions: higher detection (red), lower detection (blue), and undetected proteins (gray). Proteins detected under control are represented with a gray background. Nha1 and Pmr1 are each represented by multiple gene IDs, reflecting the variability across *R. toruloides* different genomes.

Our experimental results suggest that inorganic nitrogen metabolism is associated with the acidification of the culture environment and simultaneous EPS production (Fig. 5D). A comparison between non-buffered and buffered media containing inorganic nitrogen indicates that EPS is produced when the final pH is around or below 2, which is observed in the former but not in the latter condition (Fig. 5). Moreover, organic nitrogen-containing cultures do not sufficiently acidify the media, and EPS is not detected. This suggests that *R. toruloides* assimilates inorganic and organic nitrogen sources through distinct mechanisms, as reported for other yeasts, where nitrogen metabolism is linked to intracellular pH regulation (31, 76). Therefore, to unravel the pH maintenance processes in *R. toruloides* CCT0783, we performed a bioinformatics analysis of organellar and cellular membrane proton/ion and nitrogen transporters that are likely to contribute to pH regulation (Fig. 7). In our study, first, three cation/H^+^ antiporters were identified to regulate intracellular pH in yeast, namely, Nha1, Kha1, and Nhx1 (81), where Kha1 is localized in the membrane of the Golgi apparatus (82). Second, low pH appears to increase the gene expression of ATP synthesis in an acidic environment (83). The main regulator of pH in *S. cerevisiae* and other fungi is the P-type H-ATPase Pma1, which is present in the plasma membrane and is responsible for pumping protons outside of cells, acidifying the culture environment (84, 85). Further, V-type H-ATPases Vph1 and Stv1 are localized at the vacuolar membrane and between the Golgi apparatus and endosomes, contributing to pH maintenance (86, 87). We identified these genes in the CCT0783 strain, exhibiting 91% to 100% nucleotide similarity to those in other *R. toruloides* strains (Table S6). The proton/cation anti-transporters, except for the organellar transporters in vacuoles and mitochondria, were detected in low abundance or remained unidentified in EPS-inducing and control conditions in previous studies (Table S9). This could be attributed to the expected low abundance of membrane-bound proteins due to methodology limitations (88). Interestingly, the organellar transporters Mdm38 and Vnx1 were not present in the data of the reference NP11 strain in a previous study (8). However, one of the V-type H-ATPases (2,783/3,181) was highly abundant and upregulated during EPS-inducing conditions in earlier research (7, 46, 68) (Table S9), indicating their role in the metabolism of *R. toruloides*. In yeast, protein kinase Sch9 (10,750) has been shown to regulate V-ATPase assembly and disassembly, thereby controlling pH homeostasis in response to glucose availability (89). We identified Sch9 in the genome of CCT0783 and found its presence in EPS-inducing conditions on the proteome level (Table S9). We also detected the gene sequences of Ca^2+^-ATPases Pmr1 and Ace2 in the CCT0783 genome, which have been reported to contribute to low pH adaptation in yeast (83). Additionally, we identified two homologs of Pmr1 (13,560/13,419 and 6,549/2,724) that were previously observed at the proteome level under EPS-inducing conditions (Table S9). The genome also contains gene sequences encoding transporters for inorganic (ammonium permease) and organic (urea permease) nitrogen sources, exhibiting 89% to 100% similarity to other *R. toruloides* strains. These transporters likely play distinct roles in pH regulation and the induction of extracellular biopolymer production.

Finally, membrane-bound beta-glucan synthases have been associated with both the synthesis and transport of biopolymers, and we identified gene sequences related to these in the CCT0783 genome (90) (Tables S4, S7 to S9). Based on our analyses, we mapped putative intracellular proton transport, permeases, and biopolymer transport in *R. toruloides*, which may collectively contribute to intracellular maintenance of pH, the acidification of the culture media, and EPS production (Fig. 7).

## DISCUSSION

Our study uncovers the role of nitrogen metabolism in physiological pH maintenance and association with extracellular biopolymer production in *R. toruloides* CCT0783, providing foundational results for conducting further mechanistic studies and engineering efficient biopolymer-producing cell factories. The experimental findings present insights into a biopolymer production process, chemically characterize EPS, and map the putative EPS biosynthesis and transport pathways.

Our qualitative analysis demonstrates that the produced biopolymer is mainly composed of polysaccharides, which was corroborated through FTIR and GC-MS analysis (Fig. 1 to 3). The FTIR spectrum validated typical carbohydrate peaks previously described for microbial EPS produced by other *R. toruloides* strains (43, 91) and provided clear distinctions from the substrate and biomass spectra.

The protein content assay revealed that EPS contained approximately 1.0% of proteins, indicating it is a complex biopolymer. Our results are consistent with previous reports for *Rhodotorula acheniorum* MC, in which 1.2% of the EPS was protein (39). Pavlova et al. indicate that the crude EPS from *Sporobolomyces salmonicolor* AL_1_ contained 5.3% of proteins and 0.8% upon further purification (38). Besides proteins at 0.8%, the EPS from *Rhodotorula mucilaginosa* sp. GUMS16 contained nucleic acids (0.7%) (43). Other noncarbohydrate compounds, including glycoproteins and phospholipids, can also be found in EPS due to the non-specificity of the extraction process with ethanol (39).

GC-MS analysis demonstrated that the monosaccharide repeating units of the carbohydrate fraction are glucose, mannose, galactose, xylose, and ribose (Fig. 3). We performed a comparative literature analysis of polysaccharides produced by *Rhodotorula* yeasts, which shows a wide variation in EPS composition and production amounts (Table S10). Briefly, 54 g/L of EPS was produced from *Rhodotorula bacarum* Y68 and was characterized as a pullulan (92). *Rhodotorula* sp. strain CAH2 produced 7.5 g/L of EPS with sucrose as a carbon source, and it was composed of glucose, mannose, and galactose (93). These three sugars are among the most reported components of microbial EPS in the literature, often accompanied by smaller proportions of arabinose, fucose, and rhamnose (94–96) (Table S10). Additionally, EPS from *Rhodotorula acheniorum* MC is composed of mannose (92.8%) and glucose (7.2%) (39). When cultivated in co-cultures with yogurt starters, *Rhodotorula rubra* GED10 produced EPS that consisted mainly of mannose (83%), with small amounts of glucose, galactose, arabinose, and xylose (97). In *Rhodotorula,* mannose is the most frequently reported monosaccharide in EPS, often accounting for up to 50% of the total monosaccharide content. It has been suggested that some resistance to antimicrobial substances and osmotic stress is associated with the ecological and physiological roles of EPS in yeast (39, 98).

Nevertheless, variations in strains and cultivation conditions play a significant role in determining EPS composition, structure, and, consequently, its properties. In our study, experiments using glucose or ethanol as carbon sources establish the role of glycolytic metabolism in EPS biosynthesis by *R. toruloides*. The use of the intermediates (glucose-6-phosphate and fructose-6-phosphate) of the upper part of the glycolysis pathway for EPS production is consistent with reports from other organisms, like *Lactococcus lactis* (99). The growth and EPS production profiles for *R. toruloides* in our study agree with those of *R. mucilaginosa* sp. GUMS16 and other related yeasts (43), where EPS is not observed at the beginning of cultivation. The maximum EPS production is shown during the stationary phase (Fig. 4 and 5). The variations in the C:N ratio in our study demonstrated an increase in EPS production with increasing glucose concentrations (Fig. 5A). However, this effect did not materialize when organic nitrogen sources (urea or peptone) were used (Fig. 5B and C). Furthermore, EPS production is observed when the pH approaches 2 in cultivations with an inorganic nitrogen source (Fig. 5D). No such EPS production is observed under cultivation conditions where the culture environment is buffered and adjusted to neutral pH levels, or the pH remains above 2.5, such as when using organic nitrogen sources (Fig. 5C and D). In non-buffered pH-adjusted chemically defined media, the pH decreased to approximately 2 from the initial pH values of 4.5, 6.0, and 7.0, and EPS was produced. The final DCW also slightly increased with the increase in starting pH. These observations are consistent with those of previous studies, where biomass accumulation was reported to be optimal for *Rhodotorula* yeasts in media with an initial pH of 5 to 6.5 (100, 101). Concerning EPS, Pavlova et al. explained the importance of the pH value to produce EPS by *Sporobolomyces salmonicolor* AL_1_, where the acidification of the medium during cultivation is a typical feature and a regulatory factor for EPS biosynthesis by this yeast (38). Similar results were observed for *Candida famata* (*Debaryomyces hansenii*) and *Candida guilliermondii* (*Meyerozyma guilliermondii*) strains cultivated in media containing ammonium salts, in which EPS production occurred after the media pH dropped to 2.18 and 2.78, respectively (35). Cho, Chae, and Kim reported that the maximum EPS titer (4.0 g/L) by *Rhodotorula glutinis* KCTC 7989 was achieved in the medium containing 2.0 g/L of ammonium sulfate with an initial pH of 4.0 (34). A potential mechanism of such acidification was proposed by Peña, Pardo, and Ramírez, where the consumption of ammonium salts by *Saccharomyces cerevisiae* was reported to decrease the pH of the culture environment (76).

We performed a bioinformatics analysis to gain further insights and appreciate the putative mechanisms involved in biopolymer production. This allowed us to map a potential EPS biosynthesis and transport pathway, alongside nitrogen transporters and ion exchangers involved in the pH maintenance of *R. toruloides* (Fig. 6 and 7). Demonstrating the connection between pH and EPS production, our analysis identified the P-type H-ATPase Pma1 in the genome of strain CCT0783, showing 99.33% similarity to the NP11 strain. Its presence suggests that *R. toruloides* actively exports protons to the extracellular environment to maintain internal pH homeostasis, which is also observed in other yeasts (Fig. 7; Table S9) (85), implying that the nitrogen metabolism-linked proton exchange by *R. toruloides* is a major contributor to the acidification of the culture medium.

In summary, we demonstrated how carbon and nitrogen metabolism interact to produce extracellular biopolymers in *R. toruloides*. Glycolysis supplies both energy and precursor metabolites, some of which may contribute to the synthesis of UDP and NDP-sugars, key building blocks for the biosynthesis of extracellular biopolymers (Fig. 6). In parallel, nitrogen metabolism provides amino acids, balances the electrochemical potential in the cytosol, and contributes to the maintenance of intracellular pH in this yeast (Fig. 7). When inorganic nitrogen sources, such as ammonium, are consumed, proton pumps in the yeast ensure that the intracellular pH is maintained, causing the acidification of the extracellular environment, and EPS is observed concurrently. In contrast, the assimilation of organic nitrogen sources, like urea, appears to involve different pH maintenance mechanisms that do not cause sufficient acidification of the environment nor produce EPS.

Regarding potential applications, microbial EPSs are attractive due to their versatility, renewability, biocompatibility, and unique chemical and physical properties, making them suitable for use in food, cosmetics, pharmaceuticals, and healthcare. EPS from *Rhodotorula* species has been reported as safe for food and medical use (102, 103), exhibiting antioxidant (43) and emulsifying activities (22), as well as antiviral and antitumor properties (94).

Further studies on *R. toruloides* CCT0783 and its EPS would be necessary for the development of this oleaginous yeast as an extracellular microbial biopolymer cell factory and to develop applications for these biopolymers. We envision that the prospective research may focus on the following areas: (i) systems biology investigations of EPS production could allow identification of regulatory and metabolic engineering targets for designing biopolymer properties and enhancing yields. Previously, such approaches allowed identification of mechanistic routes for enhanced lipid production in *R. toruloides* (32, 104, 105); (ii) process optimization studies could evaluate the effects of parameters such as temperature, agitation speed, aeration, and specific nutrient availability on EPS production to improve titers and yields; (iii) chemical and physical characterizations of EPS, including glycosidic linkage analysis, determination of average molecular weight, polydispersity, identification of acidic sugars using milder methods, rheological analysis, and cytotoxicity could facilitate the development of applications for these biopolymers.

In conclusion, we expect our findings to support further development of *R. toruloides* as a model chassis microbial cell factory platform, capable of producing external biopolymers alongside its established intracellular products, namely, lipids and carotenoids. This dual intracellular and extracellular production potential, which remains to be optimized, would strengthen its appeal as a biotechnology workhorse.

## SUPPLEMENTAL MATERIAL

Supplemental tables aem.00947-25-s0001.xlsx Tables S1 to S10.

ASM does not own the copyrights to Supplemental Material that may be linked to, or accessed through, an article. The authors have granted ASM a non-exclusive, world-wide license to publish the Supplemental Material files. Please contact the corresponding author directly for reuse.
